# Activation of words with phonological overlap

**DOI:** 10.3389/fpsyg.2013.00556

**Published:** 2013-08-30

**Authors:** Claudia K. Friedrich, Verena Felder, Aditi Lahiri, Carsten Eulitz

**Affiliations:** ^1^Department of Psychology, University of TübingenTübingen, Germany; ^2^Department of Linguistics, University of KonstanzKonstanz, Germany; ^3^Department of Linguistics, Philology and Phonetics, University of OxfordOxford, UK

**Keywords:** spoken word recognition, word onset priming, ERPs

## Abstract

Multiple lexical representations overlapping with the input (cohort neighbors) are temporarily activated in the listener's mental lexicon when speech unfolds in time. Activation for cohort neighbors appears to rapidly decline as soon as there is mismatch with the input. However, it is a matter of debate whether or not they are completely excluded from further processing. We recorded behavioral data and event-related brain potentials (ERPs) in auditory-visual word onset priming during a lexical decision task. As primes we used the first two syllables of spoken German words. In a carrier word condition, the primes were extracted from spoken versions of the target words (*ano-ANORAK* “anorak”). In a cohort neighbor condition, the primes were taken from words that overlap with the target word up to the second nucleus (*ana*—taken from *ANANAS* “pineapple”). Relative to a control condition, where primes and targets were unrelated, lexical decision responses for cohort neighbors were delayed. This reveals that cohort neighbors are disfavored by the decision processes at the behavioral front end. In contrast, left-anterior ERPs reflected long-lasting facilitated processing of cohort neighbors. We interpret these results as evidence for extended parallel processing of cohort neighbors. That is, in parallel to the preparation and elicitation of delayed lexical decision responses to cohort neighbors, aspects of the processing system appear to keep track of those less efficient word candidates.

## Introduction

Current theories of speech perception assume that incoming acoustic information activates a set of word candidates or a cohort of words in the mental lexicon that overlap with this input (c.f. McClelland and Elman, 1986; Zwitserlood, 1989; Norris, 1994; Gaskell and Marslen-Wilson, 1997; Norris and McQueen, 2008). If speech was always clear-cut and intelligible and if the phonological parser aimed at processing speech as parsimoniously as possible, it would be ideal to activate only those cohort neighbors that match the acoustic input in all features and remove items from the set of activated word candidates as soon as any mismatching information is available. However, in real life the speech signal is often noisy and degraded and there is high variability within and between talkers. This will often hinder a clear-cut decision for or against a proper lexical candidate. Here we propose that this dilemma might be approached by a two-fold recognition strategy. Some processing components of the speech recognition system might handle partially mismatching lexical candidates, while others might more effectively rule out co-activated alternatives in order to end up with a single percept.

Classical models of spoken word recognition differently handle co-activated lexical entries. Connectionist models, such as TRACE (McClelland and Elman, 1986) and Shortlist (Norris, 1994) incorporate inhibitory connections between lexical representations. Activated candidates inhibit each other as a function of their respective bottom-up activation, which in turn depends on their similarity with the input. The level of lexical activation that results from bottom-up activation and lateral inhibition determines which candidate is finally recognized. Instances of the Cohort model (e.g., Marslen-Wilson and Welsh, 1978; Marslen-Wilson and Warren, 1994; Marslen-Wilson, 1987, 1990), and of the neighborhood activation model (NAM; Luce, 1986; Luce and Pisoni, 1998) do not assume any interactions among activated candidates at the lexical level. They incorporate decision rules that evaluate the activation level of a particular lexical entry with respect to the activation levels of all other representations.

Results of priming studies and eye tracking studies revealed multiple activation and rapid deactivation of candidates as soon as they are disfavored by the input (see Dahan and Magnuson, 2006; McQueen, 2007 for review). For example, the spoken Dutch first half of the word captain, *kapit*- activates targets with similar onset such as *kapitein* and *kapitaal* (“captain” or “capital”; Zwitserlood, 1989; Note that words like *kapitein* and *kapitaal* with overlapping onsets will be henceforth referred to as cohort neighbors throughout the paper). However, once *kapita*- is perceived *kapitein* is no longer facilitated. Also monosyllabic spoken word primes like *buns* did neither facilitate nor inhibit lexical decisions to targets diverging in a single segment like *guns* (Cutler et al., 1999; Gaskell and Marslen-Wilson, 2001; Gow, 2001). Similarly, eye tracking pointed to rapid deactivation of cohort neighbors. For example, if participants follow the instruction *Pick up the beaker*, their eye fixations are initially biased by a picture of the cohort neighbor *beetle*, but as soon as the signal favors one of both words, fixation probability to the cohort neighbor rapidly drops (e.g., Allopenna et al., 1998; Dahan et al., 2001; Dahan and Gaskell, 2007; Reinisch et al., 2010).

Empirical evidence is not conclusive regarding the question whether or not disfavored cohort neighbors are completely excluded from further processing. Some priming studies suggested that cohort neighbors are inhibited for further processing. For example, disyllabic French prime words such as *verger* (“orchard”) inhibited responses to cohort neighbors such as *vertige* (“vertigo”; Spinelli et al., 2001; Longtin et al., 2003). Similarly, disyllabic Spanish word onset primes such as *abun—taken* from the Spanish word *abundancia* (“abundance”) inhibited responses to cohort neighbors such as *abandano* (“abandonment”; Soto-Faraco et al., 2001). However, there is an evident bias toward fixations to cohort neighbors as compared to fixations to unrelated pictures in eye tracking data (e.g., Dahan et al., 2001; Reinisch et al., 2010). Furthermore, even candidates that are not favored at the beginning but overlap somewhat later in time with a target word (e.g., *speaker* given *beaker*) receive more fixations than unrelated words (Allopenna et al., 1998). Thus, eye tracking data do not favor an interpretation of strong inhibition of less efficient lexical candidates.

Also from eye tracking data it appears that cohort neighbors are available well beyond the time where the input favors a better matching candidate. Dahan and Gaskell (2007) determined how much acoustic information listeners need to correctly identify a given Dutch word such as *koffie* (“coffee”). The recognition point was than related to fixations to a cohort neighbor of the word such as *koffer* (“suitcase”) in displays containing both neighbors. The authors found that even after the recognition point of the referent in the spoken signal, there are still more eye fixations to the cohort neighbor than to unrelated distractors. Less effective neighbors (such as *koffer* given *koffie)* are fixated well beyond the point in time where the signal favors another candidate, which is also present in the display. Dahan (2010) concluded that this is evidence for an extended time window of activation within which appropriate and less appropriate word candidates are still available for further processing.

The assumption of an extended window of activation awaits further testing with online measures recorded within a single paradigm. The previous study by Dahan and Gaskell (2007) determined recognition points via an offline gating task. Participants were asked to judge which word would be the most likely continuation of a given word onset. The guesses and the certainty for the guesses were recorded. However, both measures might not directly be related to the online recognition processes devoted to the recognition of a given word. The present study will overcome this challenge. Here we test the hypothesis of an extended window of activation by combining the recording of lexical decision responses and event-related potentials (ERPs) in cross-modal auditory-visual word onset priming. Results previously obtained with this method revealed target word inhibition at the behavioral front end (Soto-Faraco et al., 2001). Here we will test whether or not this inhibition effect characterizes all aspects of target word processing.

Formerly we have already shown that the pattern of ERP results and of behavioral data diverges in auditory-visual word onset priming. In a previous study we tested the tolerance of the recognition process to variation that does not result in a cohort neighbor, but in a pseudoword (Friedrich et al., 2008). In a carrier word condition, primes consisted of the first syllable of German target words (e.g., *gren-Grenze* “border”). In a partial overlap condition, primes were taken from pseudoword onsets that differed from the target words in the initial place of articulation (e.g., ^*^dren-Grenze, ^*^dren is not used as a first syllable of a German word). There was no behavioral facilitation for target words primed with a partially mismatching pseudoword onset. Lexical decision latencies for the partial overlap condition did not differ from an unrelated condition. This indicates that candidates that mismatch a lexical representation in an initial element are not considered by aspects of the recognition process that are related to the lexical decision response. Nevertheless, we found reduced ERP amplitudes for the partial overlap condition compared to the unrelated condition.

Two ERP deflections observed in auditory-visual word onset priming have been related to different aspects of target word processing (Friedrich et al., 2004, 2008; Friedrich, 2005; Schild et al., 2012). Amplitudes of a left-frontal effect between 300 and 400 ms with a maximum at approximately 350 ms, the P350, vary as a function of the goodness-of-fit between the prime fragments and the target words. Our working hypothesis regarding the functional role of the P350 is that it correlates with the degree of lexical activation. From a neurocognitive perspective the P350 effect shows striking similarities to the magnetoencephalographic M350 effect (for a review see Pylkkänen and Marantz, 2003) and to the P325 effect for written words (Grainger et al., 2006; Holcomb and Grainger, 2006). Both have also been related to the activation of lexical representations in word recognition. In accordance with the bi-modal interactive activation model (BIAM, Grainger and Ferrand, 1994; Grainger and Holcomb, 2009), we assume that orthographic representations of the visual target words are co-activated due to the activation of the fragment-primed phonological representations.

A bilateral posterior central negativity between 400 and 600 ms with a peak at approximately 500 ms in auditory-visual word onset priming has been set apart from the P350 deflection (Friedrich et al., 2004, 2008; Friedrich, 2005). The central negativity shows functional differentiation to the classical N400 effect. Whereas the N400 is obtained for semantic inconsistencies (for review see Kutas and Federmeier, 2011), the central negativity in word onset priming does not reflect semantic relationship between possible continuations of the prime fragments and their following target words (Scharinger and Felder, 2011). So far, only targets that overlap with their primes elicited reduced negativity compared to unrelated targets. Topography, latency and a functional relation to phonological processing of the central negativity reveal parallels with the phonological N400 effect (Praamstra et al., 1994) and with the phonological mapping negativity (Connolly and Phillips, 1994; for review see Steinhauer and Connolly, 2009), which both are discussed to be associated with predictive phonological mechanisms in language comprehension. In accordance with this previous work, we related the central negativity in word onset priming to a mechanism that builds and updates expectancies about the phonological form of the upcoming target word on the basis of the prime.

By means of lexical decision latencies and ERPs, we test the hypotheses of a window of extended activation on the one hand, and of parallel target word processing in multiple components on the other. We used a *carrier word* condition, where the primes were the onsets of the target words (*ano-ANORAK* “anorak”); and a *cohort neighbor* condition, where the primes were taken from word onset neighbors (*ana* from *Ananas* “pineapple”) differing from the targets in the nucleus of the second syllable (*ana-ANORAK*). The onsets of the cohort neighbors that we used effectively ruled out the target words. In a pilot study, none of the 10 participants completed any of the competitor word onsets with the word that was presented as target word in the partial overlap condition (see Methods section). We related the responses in the carrier and cohort word condition to an *unrelated* condition (*idi*-*ANORAK*).

If our assumption of different effects that neighbors exert on parallel operating components of the complex word recognition process holds, we should obtain differential priming effects in behavioral and ERP data. We assume that if the system is forced to decide for or against a candidate word, as it is in the lexical decision task, this decision will be prepared in parallel and functionally encapsulated with respect to other aspects of word form processing. Therefore, the formerly observed inhibition in the cohort neighbor condition (Soto-Faraco et al., 2001) might be restricted to the behavioral front end that is tested with lexical decision responses. ERP amplitudes for the cohort word condition that are in-between ERP amplitudes for the carrier word and the unrelated condition would indicate facilitated word form processing of neighbors. If ERP indices for facilitated processing of cohort neighbors are found even after the behavioral response has been made, this would be strong evidence for an extended window of word form processing that is separate from the functional networks associated with the behavioral response.

## Methods

### Participants

Participants were 16 students (8 women) from the University of Konstanz. They were all right-handed and German was their only native language. They were paid for their participation (€ 7 per hour).

### Materials

120 German words (see Appendix) were taken as target words and as carrier words for the prime fragments. These words formed 59 pairs of trisyllabic words and one quadrisyllabic pair. The first two syllables of both words in a pair were identical up to the vowel (nucleus) of the second syllable. The nuclei of the second syllable in a pair differed from each other in various features, including place of articulation, tongue height or tongue root (e.g., AN**O**rak—AN**A**nas, *anorak—pineapple*). The stress pattern of the syllables within the word was the same for both members of the pair.

#### Primes

Spoken versions of the words were taken as carrier words for the prime fragments. They were read by a male native speaker from southern Germany in a sound-attenuated chamber. Speech signals were recorded with a Sennheiser MD421U microphone and saved on a DAT-recorder (Tascam DA-P1). Stimuli were then saved digitally on a computer with a sampling rate of 44.1 kHz and a 16 bit resolution. Off-line editing was performed with Cool Edit 2000 (©Syntrillium Software Corporation, Phoenix, AZ). Cuts were made with Cool Edit at zero crossings before the transition to the next segment. Peak amplitude was normalized to 70% of the maximum value of the sample.

The prime fragments were cut out of the carrier words such that they consisted of the segmental information up to and including the nucleus of the second syllable, which was the first segment at which both words diverged. Primes from 96 of the 120 words represented the complete second syllable, as the second syllable had CV structure. For the remaining 24 words the consonant following the nucleus (coda) of the second syllable was eliminated (see Appendix). Taking stress and vowel quality into consideration, none of the extracted word onset fragments was identical to any disyllabic German word or identical to the onset of another German word than the carrier word.

In a pre-study we tested how well the extracted fragments ruled out the pair member. To this end we presented the word onsets to 10 native speakers of German (2 men, 8 women, mean age 26 years). We asked the participants to write down the complete word from which they thought each word onset originated. No participant produced the other member of the pair with the alternative nucleus for any of the word onsets. On average, 7 participants (*SD* = 3) completed a given word onset with the carrier word. That is, the word onsets appeared to effectively rule out the pair member in an offline word completion task.

#### Targets

Written versions of the words were presented as targets. In an online Corpus of written German (www.dlexdb.de) the words that we presented as targets occurred on average 595 times (*SD* = 1053). The mean logarithmic type frequency of the target words within that Corpus is 0.11 (*SD* = 0.83). The written target words have on average 3, 7 neighbors (*SD* = 3.3) if one includes German words with the same number of letters diverging only in a single letter from the target word (Coltheart et al., 1977). Besides the 120 target words, the study included the same number of pseudowords. They were constructed by altering the last syllable of the target words (e.g., Ananas > Ananaup). In this way the first two syllables of word and pseudoword targets (and consequently also their prime fragments) were identical, which is essential for having the same baseline-activation in all conditions.

### Procedure

Stimulus presentation was handled by the software Presentation 0.61® (Neurobehavioral Systems Inc., Albany, CA). Each trial started with a white fixation cross on a black ground (font size 40) in the middle of the screen. Three hundred milliseconds after its onset, a spoken word onset (the prime) was presented via loudspeakers. The cross disappeared simultaneously with the end of the prime and was replaced immediately by the visual target word (font size 25, uppercase letters). The target word stayed on the screen for 300 ms. Participants were asked to decide whether the target word was a real German word or a pseudoword. They indicated their decision by pressing one of two mouse buttons with their thumbs. Half of the participants pressed the right button for words and the left for pseudowords, the other half did the opposite. One thousand and five hundred milliseconds after their response, the next trial started. In case they had not pressed any button, the next trial started automatically 3500 ms after the onset of the visual stimulus.

Across the four blocks, the same targets and primes were used, but combined differently across trials. In the *carrier word* condition, the target was preceded by the spoken onset of the target word (e.g., *ana-Ananas*). In the *cohort neighbor* condition, the target was preceded by the spoken word onset of the pair member of the target word [e.g., *ano* (taken from *Anorak*) *-Ananas*]. The onset of the cohort neighbor differed only in the vowel of the second syllable from the target word's onset. In the *control* condition, the target was preceded by an unrelated prime fragment. We presented each target word twice in the *unrelated* condition. To this end, the onsets of another pair of words were used. For example, *Ananas* was preceded by *ide*—taken from *Ideal* (“ideal”) in one of both unrelated trials for this target word. In the other unrelated trial *Ananas* was preceded by the onset of the pair member of Ideal, namely by *idi*—taken from *Idiom* (“idiom”). Therewith, all primes were equally often presented across the experiment and the ratio of related trials (25% *carrier word* condition and 25% *cohort neighbor* condition) and unrelated trials (25% one *control trial* and 25% another *control trial*) realized per target was identical. Furthermore, all primes used in the unrelated trials served in other trials as primes in the related conditions (e.g., *ide-Ideal, idi-Ideal, idi-Idiom, ide-Idiom*). Vice versa, all primes used in the related conditions served in other trials as primes in the unrelated condition (e.g., *ana-Ideal, ano-Ideal, ana-Idiom, ano-Idiom*).

The stimuli were presented in four blocks, each consisting of 240 trials. In each block, all 120 words and 120 pseudowords were visually presented as targets. Thus, the same target word occurred once per block and four times in the whole experiment. In the first block, 60 targets were presented in the carrier word condition, 60 targets were presented in the cohort neighbor condition; 60 targets were presented in one of both control trials, and 60 targets were presented in the other control trial. The same ratio applied for the second, third and fourth block. To keep the number of analyzed trials constant across conditions, only half of the unrelated trials entered the statistical analysis for the behavioral and ERP data. One presentation of each target word was randomly chosen from one of both unrelated trials presented across the experiment. One fourth of the analyzed unrelated target words originated from one of the four blocks respectively.

The four blocks were randomized independently of each other, so that the order of the target items differed between blocks. Half of the words appeared first in a block and their related pseudowords appeared later. For the other words and pseudowords, the order of presentation was reversed within a block. This presentation order for words and pseudowords was realized for two blocks. The reversed presentation order was realized for the two other blocks. The words and pseudowords were presented in a pseudo-randomized fashion within a block so that no more than five words (or pseudowords) appeared in a sequence. Between participants, presentation order of blocks was balanced using Latin square.

### Data acquisition and analysis

**Figure 1:**
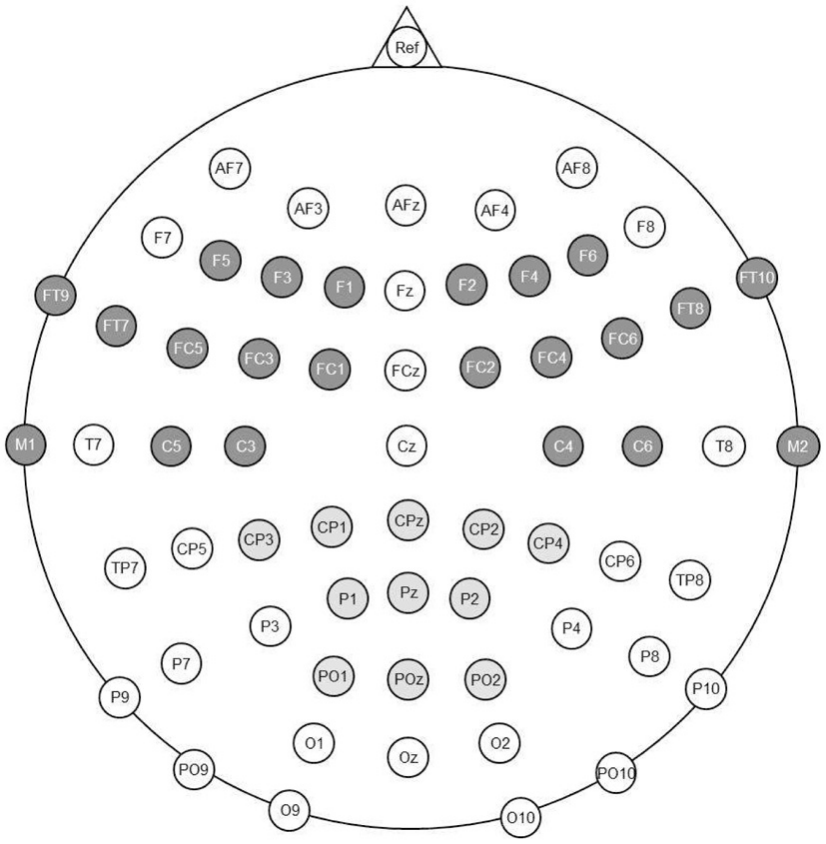
**Electrode positions recorded in the experiment**. The left and right anterior Regions of Interest are highlighted in dark gray. The central posterior Regions of Interest is highlighted in light gray.

Electrical brain activity was measured with 64 tin electrodes attached to an Elastic Cap (EASY Cap, Falk Minow Services, Herrsching-Breitbrunn, Germany). Scalp locations included 62 standard International 10–10 system locations (see Figure 1). Two additional electrodes were placed below both eyes to control for eye movements. All electrodes were online referenced to Cz. Original average reference was used for later analyses. Electrode impedances were kept below 5 kΩ. The signal was recorded with a sampling rate of 250 Hz. The EEG raw data were processed with BESA (Brain Electrical Source Analysis, MEGIS Software GmbH, Graefelfing, Germany). Blink artifacts were subtracted from the raw data. The multiple source eye correction procedure introduced by Berg and Scherg (1994) was used. Movement artifacts were rejected based on visual inspection of the continuous EEG.

EEG responses were averaged for targets with a pre-stimulus baseline of 200 ms when the fixation cross was visible on the screen. The time window of averaging was 1000 ms. Again, only those results are reported that stayed significant after application of a Greenhouse Geisser correction. Only artifact free trials with correct responses of the participants were included in the analysis of ERP data. On average 104 trials of the full overlap condition (*SD* = 11), 96 trials of the partial overlap condition (*SD* = 12) and 99 trials of the unrelated condition (*SD* = 10) entered data analysis.

## Results

Mean reaction times and percentage of correct responses are given in Tables 1 and 2. Reaction times and percentages of correct responses entered repeated measurements analyses of variance, with Condition (*carrier word* vs. *cohort neighbor* vs. *unrelated condition*) and Order (*first block* vs. *second block* vs. *third block* vs. *fourth block*) as independent variables. Normally, repetition is avoided in psycholinguistic designs; and also the former study that reported inhibition for cohort neighbors (Soto-Faraco et al., 2001) did follow this classical psycholinguistic procedure. Therefore, in addition to the analysis of effects for target words presented over all four blocks (all blocks), we also report the analysis of effects obtained for the first presentation of the target words (first block).

**Table 1:** **Mean reaction times in ms (with standard deviation) collapsed across the whole experiment (with four repetitions per target word); and separately for the first presentation (first block), first repetition (second block), second repetition (third block), and third repetition (fourth block) of the targets**.

	**Whole experiment**	**1st block**	**2nd block**	**3rd block**	**4th block**
	***M* (*SD*)**	***M* (*SD*)**	***M* (*SD*)**	***M* (*SD*)**	***M* (*SD*)**
Carrier word	603 (84)	617 (95)	598 (76)	597 (96)	598 (82)
Cohort neighbour	672 (90)	689 (114)	659 (88)	665 (83)	674 (95)
Unrelated Control	663 (86)	657 (88)	666 (93)	670 (99)	656 (75)

**Table 2:** **Mean correct responses in percent (with standard deviation) collapsed across all blocks (with four repetitions per target word); and separately for the first presentation (first block), first repetition (second block), second repetition (third block), and third repetition (fourth block) of the targets**.

	**Whole experiment**	**1st block**	**2nd block**	**3rd block**	**4th block**
	***M* (*SD*)**	***M* (*SD*)**	***M* (*SD*)**	***M* (*SD*)**	***M* (*SD*)**
Carrier word	93.0 (3.6)	91.0 (8.4)	94.0 (6.0)	93.1 (4.8)	93.8 (5.7)
Cohort neighbor	85.8 (7.6)	84.2 (8.0)	85.2 (8.9)	86.3 (10.3)	87.7 (8.0)
Unrelated control	89.0 (4.9)	86.3 (9.0)	92.1 (6.6)	89.0 (6.3)	88.8 (7.8)

### Response times

#### Whole experiment

There was a main effect of the factor *Condition*, *F*1_(2, 15)_ = 133.46, *p* < 0.001, *F*2_(2, 59)_ = 88.46, *p* < 0.001. Post-tests revealed that *carrier words* were responded to faster than *unrelated* control words, *t*1_(15)_ = 11.55, *p* < 0.001, *t*2_(59)_ = 12.38, *p* < 0.001, and also faster than *cohort neighbors*, *t*1_(15)_ = 11.03, *p* < 0.001, *t*2_(59)_ = 12.64, *p* < 0.001. There was a trend for slower responses to *cohort neighbors* compared to *unrelated words* in the by-participant analysis, *t*1_(15)_ = 2.13, *p* = 0.05, which was not confirmed in the by-item analysis, *t*2_(59)_ = 1.45, n.s. The factor *Order* did not reach significance, *F*1_(2, 15)_ and *F*2_(2, 49)_ < 1. There was a trend for an interaction of the factors *Order* and *Condition* in the by-participant analysis, *F*1_(2, 15)_ = 2.79, *p* = 0.07, which was not confirmed in the by-item analysis, *F*2_(2, 49)_ < 1.

#### First block

If only the first presentation of the target words was considered, a main effect for the factor Condition showed up as well, *F*1_(2, 15)_ = 21.09, *p* < 0.001, *F*2_(2, 59)_ = 15.54, *p* < 0.001. Post-tests revealed that *carrier words* were responded to fastest [tested against *unrelated* control words, *t*1_(15)_ = 3.68, *p* < 0.01, *t*2_(59)_ = 3.03, *p* < 0.01, and tested against *cohort neighbors*, *t*1_(15)_ = 6.47, *p* < 0.001, *t*2_(59)_ = 5.67, *p* < 0.001]. Furthermore, a robust inhibition effect for the *cohort neighbor* condition compared to the *unrelated condition* was evident, *t*1_(15)_ = 2.84, *p* = 0.01, *t*2_(59)_ = 2.65, *p* < 0.01.

### Response accuracy

#### Whole experiment

The main effect for *Condition* was significant, *F*1_(2, 15)_ = 22.74, *p* < 0.001, *F*2_(2, 59)_ = 19.14, *p* < 0.001. Responses to *carrier words* were more accurate than those to *unrelated control* words, *t*1_(15)_ = 5.30, *p* < 0.001, *t*2_(59)_ = 3.40, *p* < 0.001, and those to *cohort neighbors*, *t*1_(15)_ = 5.82, *p* < 0.001, *t*2_(59)_ = 7.15, *p* < 0.001. Responses to *cohort neighbors* were even less accurate than responses to *unrelated control* words, *t*1_(15)_ = 2.35, *p* = 0.01, *t*2_(59)_ = 2.47, *p* < 0.02. There was a trend for a main effect of the factor Order in the by-participant analysis, *F*1_(2, 15)_ = 3.28, *p* = 0.06, which was not confirmed in the by-item analysis, *F*2_(2, 59)_ = 1.89, n.s. Responses in the first block were less correct (*M* = 87.2%, *SD* = 7.2) than responses in the other blocks (2nd Block: *M* = 90.4%, *SD* = 5.3; 3rd Block: *M* = 89.4%, *SD* = 5.2; 4th Block: *M* = 90.0%, *SD* = 5.2). The interaction of the factors Condition and Order did not reach significance, *F*1_(2, 15)_ and *F*2_(2, 59)_ <1.

#### First block

A main effect for the factor Condition was found even if we considered only the first block, *F*1_(2, 15)_ = 18.59, *p* < 0.001, *F*2_(2, 59)_ = 5.40, *p* < 0.01. However, the only significant difference was a higher percentage of correct responses to *carrier words* compared *cohort neighbors*, *t*1_(15)_ = 5.94, *p* < 0.001, *t*2_(59)_ = 3.25, *p* = 0.001. Other comparisons did not reach significance, *t*1_(15)_ and *t*2_(59)_ ≤ 1.95, n.s.

### ERPs

**Figure 2:**
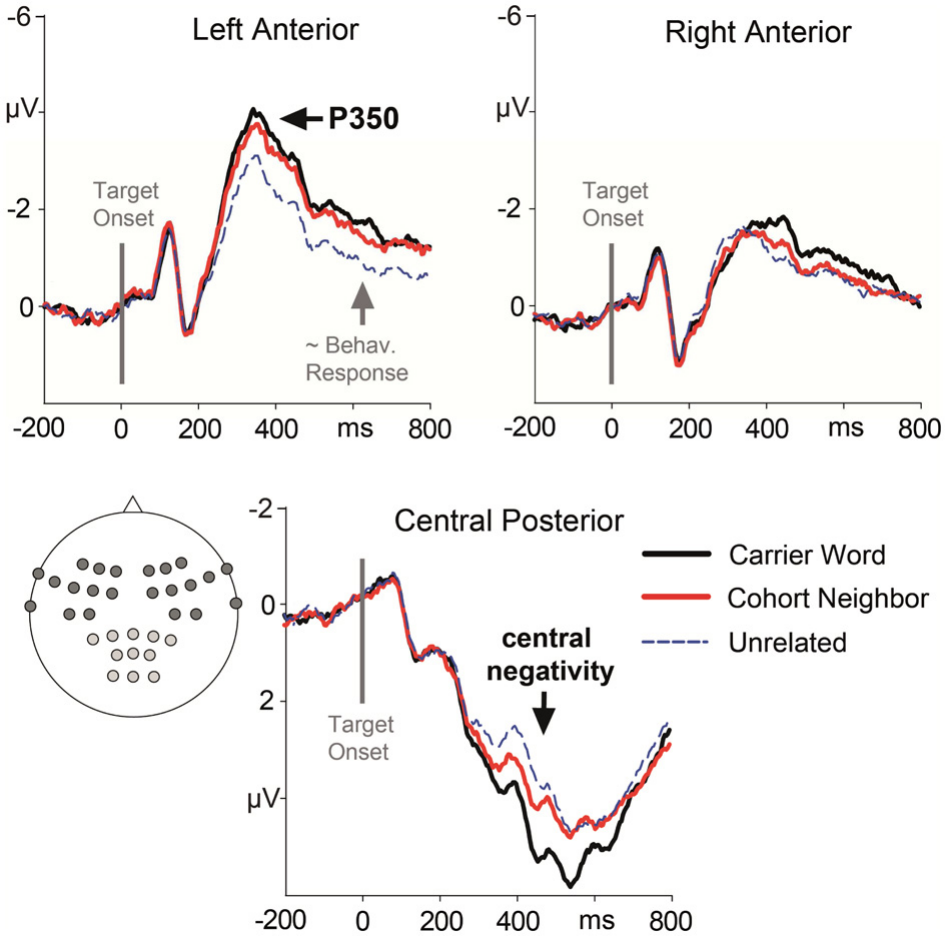
**Target-locked ERP waveforms elicited across the whole experiment with four repetitions of the target words**. ERPs are collapsed for electrode leads establishing the three Regions of Interest that entered ERP analysis. Brain responses to the carrier word condition (e.g., ano-Anorak) are given in solid black lines, to the cohort neighbor condition (ana-Anorak) in solid red lines, and to the unrelated condition (e.g., paste-Anorak) in dashed blue lines. The target word onset is indicated by a vertical line. The approximate behavioral response for the slowest condition (partial overlap) is marked by a gray arrow.

Next to the factors Condition and Order (see behavioral analysis), the additional factor Region was considered for the analyses of mean ERP amplitudes (see Figure 1). As in our previous work (Friedrich, 2005; Friedrich et al., 2008), we formed two anterior lateral Regions of Interest (ROIs), which should capture the anterior P350 effect and its left-lateralized maximum between 300 and 400 ms. In addition, we included a posterior-central ROI to capture the central negativity with a maximum between 400 and 600 ms. Each ROI included 11 electrode sites (left anterior: F1, F3, F5, FC1, FC3, FC5, FT7, FT9, C3, C5, M1; right anterior: F2, F4, F6, FC2, FC4, FC6, FT8, FT10, C4, C6, M2; central posterior: CP1, CPz, CP2, CP3, CP4, P1, Pz, P2, PO1, POz, PO2, compare Figure 1). The average target-related ERP for each ROI is depicted in Figure 2.

**Figure 3:**
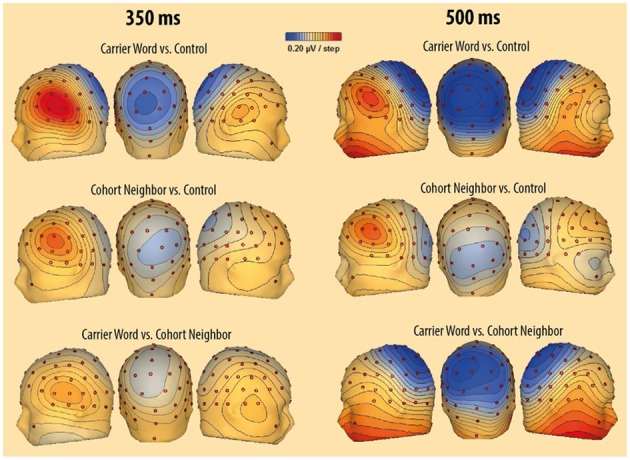
**Scalp-topography of voltage differences between the unrelated condition and the carrier word condition (above), between the unrelated condition and the cohort neighbor condition (middle), and between the cohort neighbor condition and the unrelated condition (below)**. Difference topographies are illustrated for the left side of the head (left), for the back of the head (middle) and for the right side of the head (right). The first time window (300–400 ms) is represented by ERP differences at 350 ms (left topographies). The second time window (400–600 ms) is represented by ERP differences at 500 ms (right topographies).

Also as in our earlier studies (Friedrich et al., 2004, 2008; Friedrich, 2005), a frontal left-lateralized effect (P350) and later bilateral posterior effect (central negativity) were evident. In line with our previous research, we focused on a time window ranging from 300 to 400 ms for the P350 effect, and from 400 to 600 ms for the central negativity. Representative scalp topographies of ERP differences in both time windows are depicted in Figure 3. In addition, we analyzed ERPs that follow the lexical decision responses in a time window ranging from 600 to 800 ms.

### P350 effect and starting central negativity (300–400 ms)

**Figure 4:**
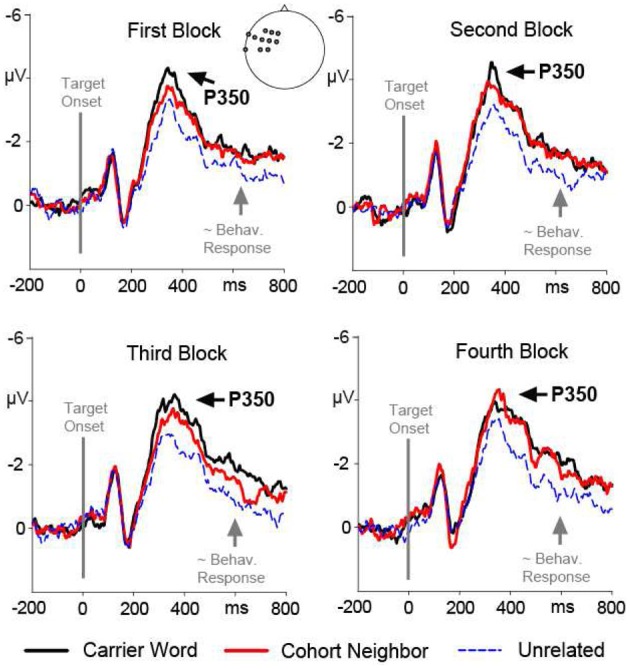
**Target-locked ERP waveforms over the left anterior ROI (P350 effect) elicited by the first, second, third and fourth presentation (block) for each target word**. Brain responses to the carrier word condition (e.g., ano-Anorak) are given in solid black lines, to the cohort neighbor condition (ana-Anorak) in solid red lines, and to the unrelated condition (e.g., paste-Anorak) in dashed blue lines. The target word onset is indicated by a vertical line. The approximate behavioral response for the slowest condition (partial overlap) is marked by an arrow.

The ANOVA revealed a significant two-way interaction of the factors Condition and Region, *F*_(2, 15)_ = 5.33; *p* = 0.01. There was no three-way interaction of the factors Condition, Region and Block, *F*_(2, 15)_ <1. Nevertheless, we included the factor Block in addition to the factor Condition in the *post-hoc* analyses for each ROI in order to rigorously test for repetition effects. A main effect of the factor Condition was evident for the left anterior ROI, *F*_(2, 15)_ = 13.85; *p* < 0.001. Amplitude differences pointed to a left-lateralized anterior P350 effect (see Figures 2, 3). For the left anterior ROI, all three conditions differed from each other. Amplitudes for the *unrelated condition* were more positive than amplitudes for the *carrier word condition*, *t*_(15)_ = 4.35; *p* < 0.001 (P350 effect). Amplitudes for the *cohort word* condition were in-between amplitudes for the *carrier word* condition, *t*_(15)_ = 2.26; *p* = 0.04, and amplitudes for the *unrelated* condition, *t*_(15)_ = 3.69; *p* < 0.001. There was only a trend for an interaction of the factors Block and Condition for the left anterior ROI, *F*_(2, 15)_ < 2.71, *p* = 0.08. For illustration purpose, mean amplitudes elicited by the three conditions over the anterior left ROI in the four blocks respectively are shown in Figure 4.

A significant effect for the factor Condition was also evident for the posterior central ROI. It indicated the beginning of the central negativity, *F*_(2, 15)_ = 9.61; *p* < 0.01. Here, the *carrier word condition* and the *cohort neighbor condition* differed from the *control condition*, *t*_(15)_ = 4.06 and *t*_(15)_ = 3.81; *p* = 0.01, respectively. The difference between the *carrier word condition* and the *cohort neighbor condition* was not significant, *t*_(15)_ = 1.59. For the posterior central ROI there was no hint for an interaction of the factors Block and Condition, *F* < 1. In sum, the cohort word condition either elicited responses in-between the carrier word condition and the cohort neighbor condition (left anterior ROI, P350 effect), or the carrier word condition was comparable to the carrier word condition (posterior central ROI, starting central negativity) in the early time window.

### Central negativity and extended P350 (400–600 ms)

The ANOVA revealed a significant interaction of the factors Condition × Region, *F*_(2, 15)_ = 16.10, *p* < 0.001. Again, there was no three-way interaction of the factors Condition, Region and Block, *F*_(2, 15)_ < 1. Nevertheless, we included the factor Block in addition to the factor Condition in the *post-hoc* analyses for each ROI in order to rigorously test for repetition effects. There was a main effect of the factor Condition for the posterior central ROI, indicating the central negativity, *F*_(2, 15)_ = 29.37; *p* < 0.001. For the posterior central ROI, amplitudes for the *carrier word* condition were less negative than amplitudes for the *unrelated* condition, *t*_(15)_ = 7.18; *p* < 0.001; and less negative than amplitudes for the *cohort neighbor* condition, *t*_(15)_ = 7.01; *p* < 0.001. There was no significant difference between the *unrelated* condition and the *cohort neighbor* condition, *t*_(15)_ = 1.14, n.s. The factors Block and Condition did not interact for the posterior central ROI.

There was also a main effect of the factor Condition for the anterior left ROI, indicating an extended P350 effect, *F*_(2, 15)_ = 13.41; *p* = 0.001. For the anterior left ROI, there was an interaction of the factors Block and Condition, *F*_(2, 15)_ = 4.73; *p* = 0.02. Condition effects were attested for the second, third and fourth block, all *F*_(2, 15)_ = 12.74, all *p* = 0.001. Across all three blocks, *post-hoc* tests indicated differences between the *carrier word* condition and the *unrelated* condition, all *t*_(15)_ = 3.61; all *p* < 0.01; as well as between the *cohort neighbor* condition and the *unrelated* condition, all *t*_(15)_ = 2.84; all *p* = 0.01. The *carrier word* condition and the *cohort neighbor* condition did not differ in any block, all *t*_(15)_ = 1.4, n.s. (compare Figure 4). In sum, robust EEG effects for the cohort word condition in the second time window were two-fold: mean amplitudes for cohort neighbors were (i) comparable to the carrier word condition over the anterior left ROI (extended P350 effect), and (ii) comparable to the unrelated condition over the central posterior ROI (central negativity).

### Post-decision ERPs (600–800 ms)

The ANOVA revealed a main effect of the factor Condition, *F*_(2, 15)_ = 7.21; *p* < 0.01. The factor Condition did neither interact with the factor Block nor with the factor Region, all *F* = 2.4, n.s. Overall, the *carrier word* condition differed from the *unrelated* condition, *t*_(15)_ = 3.09; *p* < 0.01. Also the *cohort neighbor* condition differed from the *unrelated* condition, *t*_(15)_ = 2.05; *p* = 0.05. There was no difference between the *carrier word* condition and the *cohort neighbor* condition, *t*_(15)_ = 1.4, n.s.

## Discussion

In a study with German listeners, we used spoken word onsets of cohort neighbors to prime written target words. We found that cohort neighbors inhibit lexical decision responses to their respective target words. There was a trend for inhibition as indexed by slowest and least accurate lexical decisions in the *cohort neighbor* condition compared to the *unrelated* condition over the whole experiment. Considering only responses for the first presentation of the target words, as is usual in classical psycholinguistic paradigms, we replicate inhibition of lexical decision latencies for target words preceded by disyllabic onsets of cohort neighbors (see Soto-Faraco et al., 2001). Thus, behavioral measures suggest that the system effectively eliminates or even inhibits co-activated word candidates to prepare a decision that is demanded by a psycholinguistic task at the behavioral front end.

The present ERP data show that not all aspects of processing for cohort neighbors are inhibited. In time windows preceding the delayed lexical decision responses, we obtained facilitation effects for cohort neighbors in left-anterior ERPs. Firstly, there was a gradual priming effect between 300 and 400 ms. All conditions differed from each other in P350 amplitudes. Cohort neighbors elicited mean amplitudes in-between the carrier word condition and the unrelated condition. Formerly, we related the P350 effect in this earlier time window to fine-grained lexical activation (e.g., Friedrich et al., 2004, 2008; Friedrich, 2005; Schild et al., 2012). Secondly, the left anterior effect extended into the following time window between 400 and 600 ms. In this later time window, there was no difference between cohort neighbors and carrier words, which both differed from the unrelated controls. Altogether the left-anterior P350 effect and its extension suggest that the processing of cohort neighbors is not disrupted and proceeds when a delayed behavioral response to them is being prepared.

Comparable to our former research using word onset priming, we found a central negativity with a somewhat later start and a somewhat different sensitivity to the experimental manipulation as compared to the P350 (e.g., Friedrich et al., 2004, 2008; Friedrich, 2005; Schild et al., 2012). Again, topography and latency of the central negativity parallel the phonological N400 effect (Praamstra et al., 1994) and the phonological mapping negativity (Connolly and Phillips, 1994; for review see Steinhauer and Connolly, 2009). Also in line with the N400 and the PMN, the central negativity is sensitive to the phonological relationship between prime and target word. However, the central negativity differs from the family of N400-like effects in that it has so far not been found to reflect a semantic relationship between the possible continuation of the word onset primes and the target words (Scharinger and Felder, 2011). Future research might focus more explicitly on the neural underpinnings in order to find similarities or differences between the central negativity obtained in word onset priming on the one hand; and the phonological N400 and the PMN on the other.

In the present study, there were ERP indices for blocking of cohort neighbors in the central negativity between 400 and 600 ms. Mean central posterior amplitudes did not differentiate between the cohort neighbor condition and the unrelated condition. Formerly, we related the central negativity to predictive phonological processing (e.g., Friedrich, 2005; Friedrich et al., 2008). Thus it might appear from the present results that target words that mismatch their preceding disyllabic word onsets a phoneme are excluded from predictive phonological mechanisms. Previously, we obtained reduced amplitudes of the central negativity in relation to a control condition when monosyllabic primes diverged from their targets in initial place of articulation (*dren—Grenze*, Friedrich et al., 2008; Schild et al., 2012), or in the nucleus (*kan—Konto* “account”). Together the results might indicate enhanced competition between fewer alternatives remaining for disyllabic word onset primes (present study) compared to monosyllabic word onset primes (previous studies). However, alternative explanations might consider the longer inter-stimulus interval given disyllabic compared to monosyllabic primes, or the slightly modified ROIs underlying the present analyses compared to our former work. Given the topography of the central negativity we decided to restrict the analyses on posterior central electrode leads including midline electrodes in the present work.

By combining previous and present ERP results, we conclude that neighbors exert their influence on target word processing relatively late. Our previous ERP results suggested that ERPs between 300 and 400 ms are not sensitive to the activation status of co-activated neighbors. P350 amplitudes did not vary as a function of prime length (Friedrich et al., 2004). In the present study, inhibition or blocking of cohort neighbors, which showed up in the behavioral responses and in the central negativity, was not evident in the P350 effect. Hence, ERPs in the 300 to 400 ms time window might basically reflect bottom-up activation and the goodness-of-fit between the input and the lexical representation, but not interactions among activated representations.

So far, ERP indices of co-activated neighbors in word onset priming are restricted to a late time window ranging between 400 and 600 ms. The central negativity obtained in this time window showed sensitivity to varying prime lengths in our former study. Most reduction of the central negativity was found for long primes, medium reduction for primes of medium length, and least reduction for short primes. This correlates with the fact that shorter fragments are compatible with more neighbors than longer fragments. Intriguingly, our conclusion that the later central negativity rather reflects competition effects than the earlier P350 is compatible with recent ERP research in the visual domain. Here, facilitated form-level processing for orthographic competitors appears to be indexed in an earlier N250 effect; whereas inhibited semantic processing for orthographic competitors appears to be indexed by an absent effect in the later N400 component (e.g., Holcomb et al., 2002; Massol et al., 2010).

The presently obtained diverging pattern of behavioral and neurocognitive results can be captured by the assumption of an extended window of word form processing (Dahan and Gaskell, 2007; Dahan, 2010). Particularly the left anterior ERP data and the post-decision ERPs suggest that cohort neighbors are not completely excluded from further processing. That is, some processing components of the speech recognition system appear to further handle partially mismatching candidates, while other components effectively rule out those ineffective cohort neighbors. In contrast to classical psycholinguistic models, which assume a main stream of information flow in speech recognition, there appear to be parallel operating aspects of the system. The two-fold recognition strategy that our result imply might be more easily handled by instances of the cohort model (e.g., Marslen-Wilson and Welsh, 1978, 1994; Marslen-Wilson, 1987, 1990) or versions of NAM (e.g., Luce, 1986; Luce and Pisoni, 1998), which assume that competition is resolved by a decision mechanism beyond the level of lexical activation. One would have to argue that lexical activation proceeds in parallel to this decision mechanism. In terms of connectionist models such as TRACE (McClelland and Elman, 1986) or Shortlist (Norris, 1994), which assume that competition is resolved via lateral inhibition at the lexical level, one would have to argue for a parallel track of processing inhibited words.

We are not the first who suggest that lexical decision latencies in form priming do not always reflect the activation status of a given target word in a one-to-one manner. Several authors concluded that a yes-decision in the lexical decision task rather reflects that a participant finds it likely that the target is a word than that she accesses the corresponding lexical entry (e.g., Coltheart et al., 1977; Balota and Chumbley, 1984; Grainger and Jacobs, 1996; Magnuson et al., 2001). Here we argue that lexical decisions are initiated in parallel to ongoing word form processing. With the given pattern of results it seems that inhibitory activities are prominent at the stage of response selection when the target word is in contention with co-activated alternatives. Therefore, the inhibition effect might be interpreted as a kind of by-product of the lexical decision task. Usually, the language processing system is not forced to definitely decide for or against a certain word candidate and meaning construction might proceed in parallel for several alternatives. Consequently, what we see as inhibition during lexical decision at the behavioral level might be of no relevance for online natural spoken word recognition.

Previous studies dissociating inhibition in the behavioral outcome and facilitation in neurocognitive results are compatible with the present account. For example, ERPs reflected facilitation when auditory prime words overlap in initial phonemes with auditory target words (e.g., *sad—sack*; Praamstra et al., 1994). This contrasts to the frequently obtained inhibition effect for initial overlap between primes and target words in behavioral paradigms (for reviews see Slowiaczek and Hamburger, 1992; Radeau et al., 1995; Dufour and Peereman, 2003). Similarly, the neuromagnetic M350 response, indicated facilitation for words with many phonological neighbors compared to words with fewer neighbors, even though behavioral results indicated exactly the opposite pattern (Pylkkänen et al., 2002). Similar to the present results, work with the M350 indicates that neighbors do not inhibit all aspects of spoken word processing. Interestingly enough, lateral inhibition at the lexical level has been initially sketched for a connectionist model of visual word recognition (McClelland and Rummelhart, 1981). Even for this domain, recent ERP results from a reading study rather reflect facilitated processing than inhibition of orthographic competitor words (Laszlo and Federmeier, 2009).

The assumption of a multi-component processing stream with extended activation of less efficient cohort neighbors in parallel with the initiation of a behavioral response might also help to interpret the heterogeneous results obtained from priming paradigms on the one hand and eye tracking studies on the other. Phonological priming studies either showing no facilitation (e.g., Cutler et al., 1999; Gow, 2001; Spinelli et al., 2001; Longtin et al., 2003; Friedrich et al., 2008) or inhibition for partial overlap (e.g., present study; Soto-Faraco et al., 2001) pointed to the conclusion that lexical access is “rather intolerant of any segmental mismatch” (McQueen, 2007, p.38). However, eye fixation data do not confirm this strong claim. A picture of a competitor of a spoken target word usually receives more fixations than a picture of an unrelated distractor (e.g., Allopenna et al., 1998; Dahan et al., 2001; Dahan and Gaskell, 2007). Here we argue that both measures reflect the outcome of different processing components. In parallel to the EEG data reported here, the eye fixation data might be more closely related to the status of lexical activation of a given target word than the lexical decision responses (see also Allopenna et al., 1998; Tanenhaus et al., 2000). The lexical decision responses might be dominated by inhibitory activities at the stage of response selection. The present study reveals that ERPs combined with behavioral data are a promising means to tap into different components of the complex information processing during lexical decision and to explore the role of activation and inhibition in the course of this processing in more detail within a single paradigm.

### Conflict of interest statement

The authors declare that the research was conducted in the absence of any commercial or financial relationships that could be construed as a potential conflict of interest.

## Appendix

List of the German word pairs (with English translation) used as target words and for the primes. Syllable boundaries are marked by a dash, with ambisyllabic elements given in italics. The onset of the stressed syllable is marked by an apostrophe. Primes represented the word onset up to the first phoneme that distinguished both words within a pair. The prime string that was taken from the spoken form of a respective word is bold and underlined.

**Table:**

**Carrier word**	**Carrier word**
**A-*no***-'rak (anorak)	**A-*na***-'nas (pineapple)
**Ar-gu**-'ment (argument)	**Ar-go**-'naut (argonaut)
**Di-a**-'gramm (diagram)	**Di-o**p-'trie (diopter)
**E*x-'*a**-men (exam)	**E*x*-'o**-tik (exotic)
**Fu-'ro**-re (furor)	**Fu-'ru**n-kel (furuncle)
**Ka-'ra**-ffe (carafe)	**Ka-'ro**-sse (car body)
**Ka-'ra**-te (karate)	**Ka-'ro**-tte (carott)
**Ka-ta**-'log (catalog)	**Ka-tho**-'lik (Catholic)
**Ko-'ma**n-do (command)	**Ko-'mmo**-de (commode)
**Ma-'tra**-tze (mattress)	**Ma-'tro**-se (sailor)
**Me-la**-'nom (melanoma)	**Me-lo**-'die (melody)
**Me-'ta**-pher (metaphor)	**Me-'tho**-de (method)
**Mo-nu**-'ment (monument)	**Mo-no**-'pol (monopole)
**Ok-'ta**-ve (octave)	**Ok-'to**-ber (October)
**Os-'ma**-ne (Ottoman)	**Os-'mo**-se (osmosis)
**Pa-'ra**-de (parade)	**Pa-'ro**-le (slogan)
**Pa-ra**-'sit (parasite)	**Pa-ro**-'die (parody)
**Pi-a**-'nist (pianist)	**Pi-o**-'nier (pioneer)
**Pis-'ta**-zi-e (pistachio)	**Pis-'to**-le (automatic pistol)
**Py-ra**-'mi-de (pyramid)	**Py-ro**-'ma-ne (pyromaniac)
**A-'le**-gro (allegro)	**A-'llü**-re (allure)
**An-'te**-nne (antenna)	**An-'ti**-ke (ancient world)
**I-de**-'al (ideal)	**I-di**-'om (idiom)
**Ka-'le**n-der (calendar)	**Ka-'li**-ber (caliber)
**Ka-'se**r-ne (casern)	**Ka-'si**-no (casino)
**'Ko-li**-bri (hummingbird)	**'Cho-le**-ra (cholera)
**La-'te**r-ne (lantern)	**La-'ti**-no (Latino)
**La-'ve**n-del (lavender)	**La-'wi**-ne (avalanche)
**No-'ve**-lle (novella)	**No-'vi**-ze (novice)
**Pas-'te**-te (pastry)	**Pas-'ti**-lle (pastille)
**Ak-ri**-'bie (meticulousness)	**Ak-ro**-'bat (acrobat)
**Al-'bi**-no (albino)	**Al-'ba**-ner (Albanian)
**An-'gi**-na (angina)	**An-'go**-ra (angora)
**A-ppe**-'tit (appetite)	**A-pa**-'thie (apathy)
**E-le**-'ment (element)	**E-lo**-'quenz (eloquence)
**Em-'pö**-rung (outrage)	**Em-'po**-re (gallerie)
**Fa-'ce**t-te (facet)	**Fa-'ssa**-de (face of a building)
**Fi-'ne**-sse (finesse)	**Fi-'na**-le (finale)
**Ga-'lee**-re (galley)	**Ga-'llo**-ne (gallon)
**Ga-le**-'rie (gallery)	**Ga-la**-'xie (galaxy)
**Ho-ri**-'zont (horizon)	**Ho-ro**s-'kop (horoscope)
**In-fe**k-'tion (infection)	**In-fo**r-'mant (informant)
**In-se**-'rat (insertion)	**In-so**l-'venz (insolvency)
**Ka-'bi**-ne (cabin)	**Ka-'ba**-le (cabal)
**Ka-bi**-'nett (cabinet)	**Ka-ba**-'rett (cabaret)
**Ka-'ni**n-chen (bunny)	**Ka-'no**-ne (cannon)
**Ka-'pi**-tel (chapter)	**Ka-'pu**-ze (hood)
**Ka-'the**-ter (lectern)	**Ka-'tha**r-sis (catharsis)
**Kom-me**n-'tar (comment)	**Kom-ma**n-'dant (commandant)
**Kom-pe**-'tenz (competence)	**Kom-pa**-'nie (company)
**Kon-se**-'quenz (consequence)	**Kon-so**-'nant (consonant)
**Kor-re**-'lat (correlate)	**Kor-ro**-'sion (corrosion)
**'Li-bi**-do (libido)	**'Li-ba**-non (Lebanon)
**Li-te**-'rat (litterateur)	**Li-ta**-'nei (litany)
**Ma-'ri**-ne (marine)	**Ma-'ro**-ne (chestnut)
**Me-'li**-sse (Melissa)	**Me-'lo**-ne (melon)
**Mi-ne**-'ral (mineral)	**Mi-na**-'rett (minaret)
**Mi-'ni**s-ter (minister)	**Mi-'nu**-te (minute)
**Re-li**-'gion (religion)	**Re-la**-'tion (relation)
**Ter-'ri**-ne (terrine)	**Ter-'ra**-sse (terace)
